# The Epidemiology and Management of Bell’s Palsy in the Sudan

**DOI:** 10.2174/1874210601812010827

**Published:** 2018-10-25

**Authors:** Ahmed Hassan Kamil Mustafa, Ahmed Mohammed Sulaiman

**Affiliations:** Department of Oral And Maxillofacial Surgery, University of Khartoum, Khartoum, Sudan

**Keywords:** Bell's palsy, Facial nerve paralysis, Lower motor neuron, Degrees of sequelae, Sudanese patients, Winter season

## Abstract

**Background:**

Bell’s palsy is an acute idiopathic facial nerve paralysis of sudden onset. It is the most common cause of lower motor neuron facial nerve paralysis with an annual incidence of 15-30 per 100,000.

The objective of this work is to study the prevalence and the management of Bell’s palsy in the Sudan. A descreptive retrospective cross-sectional study was carried at Khartoum Teaching Dental Hospital, Khartoum General Teaching Hospital.

In the retrospective, the records and files of 698 patients with Bell’s palsy, were reviewed in relation to age, gender, site, risk factors, season, and type of treatment.

In addition, 48 patients with Bell’s palsy were evaluated using the House–Brackman scale in relation to the above-mentioned variables.

Therefore, a total number of 746 cases were studied. Fifty five percent of them were females and the remaining 45% were males, around 38% of them were in the age group 21-40 year. Fifty seven percent of the patients were affected on the right side of the face. Winter was the commonest season of onset where 53.5% of the cases occurred. Steroids are the commonly prescribed drugs in majority of the cases, accounting for 47.3%.

**Study Design:**

The study is a retrospective cross sectional hospital based study. The study was carried out in Khartoum Teaching Dental Hospital and in the Physiotherapy Department of Khartoum Teaching General hospital.

The files and records of the patients with Bell’s palsy in Khartoum Teaching Dental Hospital in the years 1/1/2004 -31/12/2008, and Khartoum Teaching General Hospital (physiotherapy department) in the years 2007- July 2009 (total number 746).

**Results:**

A total number of 746 cases were studied . Fifty five percent of them were females and the remaining 45% were male. Around 38% of them were in the group 21-40 year. Fifty seven percent of the patients were affected on the right side of the face. Winter was the commonest season of the onset where 53.5% of the cases occurred.

**Conclusion:**

The study showed predominance of females. A peak incidence was seen in the age group 21-40 years. A predilection was found for the right side of face.

## 1 INTRODUCTION AND BACKGROUND

Bell’s palsy is an acute idiopathic peripheral facial nerve paralysis of sudden onset. It is the most common cause of lower motor neuron facial nerve paralysis [1].

Facial nerve paralysis has been known since ancient times by the Egyptians, Greeks, Romans, Incas and other native cultures [2].The oldest artistic representation of facial nerve paralysis is a clay head from Egypt (4,000 years old), showing a right peripheral facial nerve paralysis.

In the Middle Ages and during the Renaissance, many artists portrayed figures with asymmetrical and distorted faces. The portrait of Mona Lisa is the most famous and her enigmatic smile has been discussed for many years all over the world and at several facial nerve symposiums.

The first medical studies of the disease should be attributed to Avicenna [3]. He was the first to record the differences between central and peripheral facial paralysis.

Although the name of Sir Charles Bell, who published his findings in 1821, is usually associated with this condition, there are two papers, one published by Niclaus A. Friedrich in 1798, and the other by Richard Powell in 1813, whose observation of onset, physical findings, natural history and recovery preceded those of Charles Bell [4].

Acute idiopathic peripheral facial palsy is a common disease with an annual incidence of 15-30 per 100,000 population [5]. Most patients recover completely, but about 15-30% are reported to be left with different degrees of sequelae [5, 6].

There are many possible causes of Bell’s palsy, but still the aetiology remains obscure [5]. The term Bell’s palsy should be restricted to idiopathic facial palsy. It accounts for 60-70% of all cases of unilateral facial paralysis [1].

Very little has been published on the incidence of Bell’s palsy for various reasons. Physicians from many specialties see and treat patients with Bell’s palsy; and some patients with the disorder do not seek treatment because it is painless and because the paralysis may be limited, or of short duration [7].

The epidemiology of acute idiopathic peripheral facial palsy has been discussed in several articles, sometimes with contradictory findings [8].

In Sudan, no study has been done on the epidemiology, the course, treatment and recovery of Bell’s palsy.

The only published study was a case report of Bell’s palsy in seven Sudanese children, by Abbas and Prabhu in 1981 [9].

The purpose of this study is to study the course and treatment of Bell’s palsy among Sudanese patients.

### 1.1 Epidemiology

Acute peripheral facial palsy is a common disease with an annual incidence of 15-30 per 100,000 population [5, 10]. The incidence increases with age [11]. Familial inheritance has been found in 4-14% of cases [12]. The incidence of Bell’s palsy reaches a maximum between the ages 15- 45 years, the disease is significantly less common below the age of 15 years and above the age of 60 [6, 12]. Both sexes are affected equally [13]. Pietersen found that both sides of the face are affected equally, although in other studies a predilection for the left side was found [6]. In many studies, no seasonal variation was found [6, 13]. De Diego *et al.*, concluded that seasonal trend in the incidence of Bell’s palsy does exist (Winter season), with few cases occurring in summer [14].

### 1.2 Risk Factors

Hilsinger *et al*., demonstrated a calculated frequency of Bell’s palsy in the pregnant female 3.3 times that of non-pregnant females of the same age (45.1 *vs* 17.4 cases per100.000 birth per year, respectively) [15]. For women in the third trimester of pregnancy in particular, the frequency rose to 118.2 cases per 100.000 births per year [15]. Grant *et al*, suggested a significantly greater likelihood of an unsatisfactory recovery compared with the non-pregnant population [16]. Generalized tissue edema in pregnancy causing mechanical compression of the facial nerve in the fallopian canal might be responsible for the increased frequency in pregnancy [16].

Paolino *et al.*, reported a greater frequency of arterial hypertension and lipid disorders in patients with Bell’s palsy than in controls [7, 17]. Pressure changes in the circulation of patients, especially diastolic hypertension, may disturb the delicate balance of the pressure systems existing inside the facial canal, leading to impairment of the intrafunicular circulation, and subsequently to nerve damage [17].

The risk of Bell’s palsy is increased in diabetes [18]. A suggestion was made that Bell’s palsy in some diabetics might be resulting from ischemia of the facial nerve, due to diabetic small vessel disease [19]. Pecket *et al*., proposed that sparing of taste fibres in cases of concurrent diabetes and Bell’s palsy may be related to diabetic small vessel disease, and that these cases of Bell’s palsy may in fact be regarded as a diabetic mononeuropathy [19].

### 1.3 Objectives

To study the course and treatment of Bell’s palsy among Sudanese patients.

## 2 PATIENTS AND METHODS

### 2.1 Study Design

The study is a retrospective cross sectional hospital based study. The study was carried out in Khartoum Teaching Dental Hospital and in the Physiotherapy Department of Khartoum Teaching General hospital.

The files and records of patients with Bell’s palsy in Khartoum Teaching Dental Hospital in the years 1/1/2004 -31/12/2008, and Khartoum Teaching General Hospital (physiotherapy department) in the years 2007-July 2009 (total number 746) were reviewed in relation to:

1. Age
2. Gender
3. Site
4. Risk factor
5. Season
6. Type of treatment

### 2.2 Inclusion Criteria

Patients affected by acute onset facial paralysis without detectable cause.

### 2.3 Exclusion Criteria

- Known traumatic, inflammatory and neoplastic, pathology of the facial nerve in its intra or extracranial course.
- Bilateral facial paralysis.
- Concurrent disease of central or peripheral nervous system.

### 2.4 Data Analysis

The data was analysed using Statistical Package of Social Sciences (*SPSS*) version 15.

## 3 RESULTS

The question of a gender predominance among patients having Bell’s palsy has been discussed in the majority of the published reports if not all of them Table **1**. In this study, the total number of patients was (n= 746) patients. Fifty five percent of the patient were females showing clearly a predominance of females and that the difference is statistically significant (*p* = 0.007).

Table **2**, shows a peak incidence in the group 21-40 years accounting for 37.8% of the series, which is statistically significant (*p*=.001).

From the total number of patients the study 57.2% of patients were affected on the right side of the face. The study showed that the right side is predominantly affected, and that the predilection for this side is statistically significant (*p*=.001) which was illustrated in Table **3**.

On correlating the gender with the side (Table **4**), there is no influence of the gender on the side affected. On the other hand, as can be seen from Table **5** there is also noinfluence of the age group or the gender on the side affected by Bell’s palsy.

Table **6** showed that, around 54 percent of our patients caught the disease in winter season indicating that there is peak clustering of Bell’s palsy in winter season. This is followed by Autumn in which, 35.5% of our patients had the disease and the least number of cases (11%) were seen in Summer.

In the present study, around 8% of our patients were suffering from hypertension as shown in Table **7**. and the relation between it and Bells’ palsy appears to be statistically significant.

As shown in Table **8**, out of 746 patients with Bell’s palsy 10.7% were found to be diabetics.

As shown in Tables **9** and **10**, the total number of females in the study is (n=410) patients. Of these, 67.3% of the patients are in the age of pregnancy years of this group, 43.4% of the patients are married and in the pregnancy age. The percentage of pregnant females (n=58) from the total number of married women and in the pregnancy age is 32.5% which is a high percentage, suggesting a strong association between Bell’s palsy and pregnancy, as illustrated in Table **8**.

47.3% of the patients received steroid therapy alone, 31.1% received a combination of steroids and physiotherapy, 15.8 % received physiotherapy only, and the remaining patients (5.8%) received other treatments which include vit B12, antiviral drugs, and non-steroidal anti- inflammatory drugs, as shown in Tables **11** and **12**.

## 4 DISCUSSION

Not much is published on the epidemiology of Bell’s palsy and in the dearth of literature published on it contradicting results can be seen. In this study, a total number of 746 of cases of Bell’s palsy were studied. Of these, 746 cases are records of patients seen in the period 2004 -2008 in Khartoum Teaching Dental Hospital, and at Khartoum General Teaching Hospital (Physiotherapy Department) in the period from Jan 2007 to July 2009.

### 4.1 Distribution

#### 4.1.1 Gender

As shown in Table **1** the question of a gender predominance among patients having Bell’s palsy has been discussed in the majority of the published reports if not all of them. In this study, the total number of patients was (n= 746) patients. Fifty five percent of the patient were females showing clearly a predominance of females and that the difference is statistically significant (*p* = 0.007). Similar figures were reported by Valenca *et al*., from Portugal [20] ^,^where the female gender comprised 66.7% of the cases. Our finding in this respect are also in agreement with the figures shown by Goncalves *et al*., in Brazil [21], who reported a predominance of female mounting up to 77.71% of the cases.

#### 4.1.2 Age

This subject has also been debated, without reaching a definitive conclusion.

Table **2**, shows a peak incidence in the group 21-40 years accounting for 37.8% of the series, which is statistically significant (*p*=.001) . The incidence of Bell’s palsy shows a decline below the age of 20 years 23.8% (n=175) patients, and above the age of 61years, (10.2%)) (n=76) patients.

This study demonstrated convincingly the influence of age on the incidence of Bell’s palsy. Pietersen from Denmark [6] reported similar results, and found the incidence of Bell’s palsy reaches a maximum between the ages of 15 and 45 years. The author also found that Bell’s palsy is significantly less below the age of 15 years as well as above the age of 60 years. Goncalves *et al*., from Brazil [21] showed similar findings and that the incidence of Bell’s palsy is high in the age group bracket 31-60 years and low above the age of 60 years.

#### 4.1.3 Site

From the total number of patients the study 57.2% of patients were affected on the right side of the face. The study showed that the right side is predominantly affected, and that the predilection for this side is statistically significant (*p*=.001) which was illustrated in Table **3**. Because the sample size is small we can’t assume this result representing the actual situation. Further investigation is needed to verify or negate this observation. Nevertheless, our findings are similar to those reported by Savettieri *et al*., from Italy [22] who found that the right side was affected in 63% of their cases.

On correlating the gender with the side (Table **4**), there is no influence of the gender on the side affected. On the other hand, as can be seen from Table **5** there is also no influence of the age group or the gender on the side affected by Bell’s palsy. These finding are in accordance with findings of Pietersen [6] from Denmark, Katusic *et al*., from the United states [7]. Diego *et al*., from Spain [23] in their study found no side predilection and did not demonstrate any effect of gender or age or on the side involved in Bell’s palsy.

#### 4.1.4 Season

The seasonal variation of Bell’s palsy is debatable, there are contradicting views in the literature about this topic.

Table **6** showed that, around 54 percent of our patients caught the disease in winter season indicating that there is peak clustering of Bell’s palsy in winter season. This is followed by Autumn in which, 35.5% of our patients had the disease and the least number of cases (11%) were seen in Summer.

The findings of our study showed a seasonal clustering of cases in Winter. This is in accordance with the findings of Goncalves *et al*., from Brazil [21] which showed a predominance of cases (31.38%) in winter. The same result was found by De Diego *et al*., Spain [23] who reported seasonal clustering in Winter with fewer cases occurring in summer. On the other hand, Pietersen [6] from Denmark, and Rowland *et al*., from UK [13] did not demonstrate any seasonal variation with Bell’s palsy.

### 4.2 Risk Factors

#### 4.2.1 Hypertension

Holland, Devriese *et al*., [24] in a sample of 1235 patients with Bell’s palsy found that 13% of them had hypertension. Katusic *et al*., [7], supported the hypothesis that hypertension is a risk factor for Bell’s palsy and also concluded that increasing age resulted in a greater frequency of incomplete recovery.

The relation of Bell’s palsy and some systemic diseases that are prevalent in the community need to be addressed more scientifically. The studies that associate the incidence of Bell’s palsy with some of these systemic diseases, none has mentioned the issue of the prevalence of these diseases in the community concerned.

In Sudan, hypertension as well as diabetes are now common diseases with a prevalence ranging from 18% and 20%, respectively. In the present study, around 8% of our patients were suffering from hypertension as shown in Table **7** and the relation between it and Bells’ palsy appears to be statistically significant. Nonetheless, it is difficult to assume that the patients with hypertension are more prone to have Bell’s palsy than their normal counterparts.

#### 4.2.2 Diabetes Mellitus

Many studies have reported an association between the incidence of Bell’s palsy and diabetes. Yazdi *et al*., from Iran [18] found increased incidence of Bell’s palsy in patients with diabetes, and Pecket *et al*., from Israel [19] demonstrated a strong association between Bell’s palsy and diabetes. Some of these authors suggested that the small vessels disease is associated with diabetes mellitus as a cause of nerve ischemia leading to Bell’s palsy. Interestingly, none of them mentioned anything about the prevalence of the disease in their countries and its correlation with that associated with Bell’s palsy.

As shown in Table **8**, out of 746 patients with Bell’s palsy 10.7% were found to be diabetics. The prevalence of diabetes mellitus in the Sudan is 20% percent. When we correlate this percentage to the percentage of the diabetics among our Bell’s palsy patients, it would not be logical to assume that diabetes mellitus is a risk factor for Bell’s palsy.

#### 4.2.3 Pregnancy

As shown in Tables **9** and **10**, the total number of females in the study is (n=410) patients. Of these, 67.3% of the patients are in the age of pregnancy years of this group, 43.4% of the patients are married and in the pregnancy age. The percentage of pregnant females (n=58) from the total number of married women and in the pregnancy age is 32.5% which is a high percentage, suggesting a strong association between Bell’s palsy and pregnancy, as illustrated in Table **8**.

These findings in our study are in accordance with the findings of many investigators. Hilsinger *et al*., [15], from the USA found a greater frequency of Bell’s palsy in pregnant females 3.3 times that of non-pregnant. Grant *et al*., [16], from USA also reported a similar result and added that there was a greater likelihood of unsatisfactory recovery in pregnant patients.

The increased prevalence of Bell’s palsy in pregnant females may be due to gestational immune suppression resulting from the rising cortisol levels in pregnant women. This might induce reactivation of dormant or inhabitant viruses in the body [15]. Generalized tissue odema associated with pregnancy causing mechanical compression of the facial nerve in the fallopian canal is another possibility. Associations with higher level of progesterone and oestrogen in pregnancy, and toxaemia of pregnancy have been suggested as a cause.

### 4.3 III Type of Treatment

47.3% of the patients received steroid therapy alone, 31.1% received a combination of steroids and physiotherapy, 15.8% received physiotherapy only, and the remaining patients (5.8%) received other treatments which include vit B12, antiviral drugs, and non-steroidal anti- inflammatory drugs, as shown in Tables **11** and **12**.

Generally, Bell’s palsy should be considered an emergency that should be treated using all available measures against possible multiple factors [6]. The early use of steroids give better result and according to Ramsey *et al*., [25] corticosteroid treatment provides a clinically and statistically significant improvement in recovery and function of the nerve if given within the first 72 hours.

## CONCLUSION

Females are more susceptible to Bell’s palsy than the males in the Sudan.

The age group 21- 40 was the commonest age group involved in Bell’s palsy.

There is a predilection of Bell’s palsy for the right side of the face, with no influence of gender or ages observed.

There is an association between Bell’s palsy and pregnancy and Winter is the commonest season of the onset. It is difficult to draw an association between Bell’s palsy with hypertension and diabetes mellitus, as both diseases are prevalent with high frequencies in the Sudan.

The treatment of choice of Bell’s palsy is a combination therapy that includes steroids.

## RECOMMENDATIONS

A large population-based epidemiological study of Bell’s palsy is needed in Sudan to determine the prevalence of the disease.

## Figures and Tables

**Table 1:** Distribution of Bell’s palsy in KTDH and in KTH according to gender.

Gender*	**Frequency**	**Percentage%**
**Male** **Female** **Total**	336 410 746	45% 55% 100%

*According to *Chi*-Square test, the *p* value was 0.007.

**Table 2:** Distribution of Bell’s palsy in KTDH and in KTH according to age group.

Age Group*	**Frequency**	**Percentage%**
0 -20 21 -40 41-60 61 -80 Total	178 282 210 76 746	23.8 37.8 28.2 10.2 100

* According to *Chi*-Square test, the *p* value was 0.001.

**Table 3:** Distribution of Bell’s palsy in KTDH and in KTH according to site.

Site*	**Frequency**	**Percentage%**
Right Left Total	427 319 746	57.2 42.8% 100

*According to *Chi*-Square test, the *p* value was 0.001.

**Table 4:** Correlation between gender and side affected among patients with Bell’s palsy in KTDH and KTH.

**-**	**-**	**The Side**			**Total**
**Gender**	**-**	**Right**		**Left**	
Male	Count	188		155	336
	% within Gender	53.9%		46.1%	100.0%
	% within The Side	42.4%		48.4%	45.0%
Female	Count	246		164	410
	% within Gender	60.0%		40.0%	100.0%
	% within The Side	57.6%		51.4%	55.0%
Total	Count	427		319	746
	% within Gender	57.2%		42.8%	100.0%
	% within The Side	100.0%		100.0%	100.0%
*Chi*-Square Test					
-		Gender*side			
Chi-square		2.836			
Aymp.sig.		.092			

**Table 5:** Correlation between age group and side affected among patients with Bell’s palsy in KTDH and KTH.

**-**		**The Side**			**Total**
**Age Group**	**-**	**Right**		**Left**	
0-20	Count	117		61	178
	% within Gender	65.7%		34.3%	100.0%
	% within The Side	27.4%		19.1%	23.9%
21-40	Count	154		128	282
	% within Gender	54.6%		45.4%	100.0%
	% within The Side	36.1%		40.1%	37.8%
41-60	Count	112		98	210
	% within Gender	53.5%		46.7%	100.0%
	% within The Side	26.2%		30.7%	28.2%
61-80	Count	44		32	76
	% within Gender	57.9%		42.1%	100.0%
	% within The Side	10.3%		10.0%	10.2%
**Total**	Count	427		319	746
	% within Gender	57.2%		42.8%	100.0%
	% within The Side	100.0%		100.0%	100.0%
*Chi*-Square Test					
-		Age group*side			
Chi-square		7.362			
Asymp.sig.		.061			

**Table 6:** Distribution of Bell’s palsy in KTDH and in KTH according to season.

Season*	**Frequency**	**Percentage%**
Autumn Summer Winter Total	265 82 399 746	35.5 11 53.5 100

*According to Chi-Square test, the *p* value was 0.001.

**Table 7:** Hypertension among patients of Bell’s palsy in KTDH and in KTH.

Hypertension*	**Frequency**	**Percentage%**
Yes No Total	63 683 746	8.4 91.6 100

*According to *Chi*-Square test, the *p* value was 0.001.

**Table 8:** Diabetes mellitus among patients of Bell’s palsy in KTDH and in KTH.

Diabetes Mellitus*	Frequency	Percentage%
Yes No Total	80 666 746	10.7 89.3 100

*According to *Chi*-Square test, the *p* value was 0.001.

**Table 9:** Pregnancy among patients with Bell’s Palsy in KTDH and KTH.

Pregnancy*	**Frequency**	**Percentage%**
Yes No Total Missing system Total	58 352 410 336 746	14.2 85.8 55 45 100

*According to *Chi*-Square test, the *p* value was 0.001.

**Table 10:** Distribution of Bell’s Palsy among female patients of KTDH and KTH according to fertility and marital status.

-	**Frequency**	**Percentage%**
(1 -14)years Fertile age(15 -49)years Married and in pregnancy age Married and pregnant Total married Menopausal age (>51years) Total	44 276 178 58 266 90 410	10.7 67.3 43.4(from total) 32.5 64.9(from total) 21.9(from total) 100

**Table 11:** Treatments received by patients with Bell’s Palsy in KTDH and KTH.

**Treatment**	**Frequency**	**Percentage%**
Steroids Physiotherapy Steroids+physiotherapy Other treatments Total	330 110 217 41 698	47.3 15.8 31.1 5.8 100

**Table 12:** types of other treatments received by patients with Bell’s palsy in KTDH and KTH

Other Treatments	Frequency	Percentage %
Vit B12	19	46.3
Antiviral	13	31.7
Anti-inflammatory(NSAID)	9	22
Total	41	100
